# What Is the Place of Intermediate Care Unit in Patients with COVID-19? A Single Center Experience

**DOI:** 10.1155/2023/8545431

**Published:** 2023-04-20

**Authors:** Hale Bülbül, Gözde Derviş Hakim, Cengiz Ceylan, Murat Aysin, Şükran Köse

**Affiliations:** ^1^Hematology Department, Health Sciences University İzmir Medicine Faculty, Tepecik Training and Research Hospital, Yenişehir, Konak, İzmir, Turkey; ^2^Gastroenterology Department, Health Sciences University İzmir Medicine Faculty, Tepecik Training and Research Hospital, Yenişehir, Konak, İzmir, Turkey; ^3^Public Health Department, İzmir Katip Çelebi University Atatürk Training and Research Hospital, Karabağlar, İzmir, Turkey; ^4^Infectious Diseases Department, Health Sciences University İzmir Medicine Faculty, Tepecik Training and Research Hospital, Yenişehir, Konak, İzmir, Turkey

## Abstract

**Introduction:**

COVID-19 pandemic has led to an increased rate of intensive care unit (ICU) stays. Intermediate care units (IMCUs) are a useful resource for the management of patients with severe COVID-19 that do not require ICU admission. In this research, we aimed to determine survival outcomes and parameters predicting mortality in patients who have been admitted to IMCU.

**Materials and Methods:**

Patients who were admitted to IMCU between April 2019 and January 2021 were analyzed retrospectively. Sociodemographics, clinical characteristics, and blood parameters on admission were compared between the patients who died in IMCU and the others. Blood parameters at discharge were compared between survived and deceased individuals. Survival analysis was performed via Kaplan–Meier analysis. Blood parameters predicting mortality were determined by univariate and multivariate Cox regression analysis.

**Results:**

A total of 140 patients were included within the scope of this study. The median age was 72.5 years, and 77 (55%) of them were male and 63 (45%) of them were female. A total of 37 (26.4%) patients deceased in IMCU, and 40 patients (28.5%) were transferred to ICU. Higher platelet count (HR 3.454; 95% CI 1.383–8.625; *p*=0.008), procalcitonin levels (HR 3.083; 95% CI 1.158–8.206; *p*=0.024), and lower oxygen saturation (HR 4.121; 95% CI 2.018–8.414; *p* < 0.001) were associated with an increased risk of mortality in IMCU. At discharge from IMCU, higher procalcitonin levels (HR 2.809; 95% CI 1.216–6.487; *p*=0.016), lower platelet count (HR 2.269; 95% CI 1.012–5.085; *p*=0.047), and noninvasive mechanic ventilation requirement (HR 2.363; 95% CI 1.201–4.651; *p*=0.013) were associated with an increased risk of mortality. Median OS was found as 41 days. The overall survival rate was found 40% while the IMCU survival rate was 73.6%.

**Conclusions:**

IMCU seems to have a positive effect on survival in patients with severe COVID-19 infection. Close monitoring of these parameters and early intervention may improve survival rates and outcomes.

## 1. Introduction

The consequences of coronavirus disease 2019 (COVID-19) caused by severe acute respiratory syndrome coronavirus 2 (SARS-CoV-2) which has occurred in the Wuhan region of China and spread all over the world and have influenced all stages of health services [1]. The severity of patients infected with COVID-19 could be elaborated as follows: 15% severe illness and 5% have critical illness. Overall mortality ranged from 0.25% to 3.0%. Case fatality rates are much higher for vulnerable populations, such as patients over the age of 80 years (>14%) and those with comorbid conditions (10% for those with cardiovascular disease and 7% for those with diabetes) [2, 3]. Many countries do not have sufficient ICU bed capacity and prolonged hospitalization of patients who require invasive mechanic ventilation [4, 5]. Intermediate care units (IMCUs) are logistically located between the hospital ward and the ICU. These units provide continuous monitoring, noninvasive respiratory support, and vasopressors and are increasingly utilized for their potential to optimise hospital productivity and reduce pressure on ICU beds and ICU mortality [6, 7]. Up to date, some indicators such as hypersensitive C-reactive protein (h-CRP), procalcitonin (PCT), creatinine, LDH, aspartate aminotransferase (AST), hypersensitive cardiac troponin-I, prothrombin time, D-dimer, and lymphocyte count have provided supportive information to assess the severity of the disease and predict the prognosis of COVID-19 [8–10]. In this research, we aimed to determine survival outcomes and parameters predicting mortality in patients who have been admitted to IMCU.

## 2. Materials and Methods

### 2.1. Patients

All patients underwent chest CT and RT-PCR tests before admission to IMCU. Individuals who were >18 years old, with a positive RT-PCR result that was hospitalized in the IMCU patients were included in this study. Patients <18 years of age, having pregnancy, missing baseline, or outcome data, and with negative RT-PCR results were excluded. In the first step, the patients were divided into two groups of individuals who were deceased in IMCU and others. Patients who were discharged from IMCU were allocated to two groups survived and deceased subjects.

### 2.2. Method

**Figure 1:**
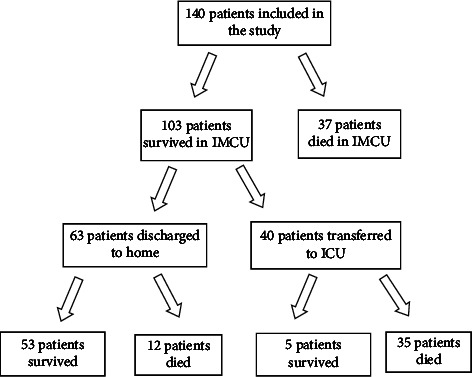
Flowchart of the study. IMCU, intermediate care unit; ICU, intensive care unit.

Following the approval of the study protocol by the Ministry of Health (2020-06-05T19_28_13. Xxml), ethics committee approval of the study from Tepecik Training and Research Hospital was obtained (Date 25.01.2021 No: 2021/01-22). The study was performed in the IMCU Department of Tepecik Training and Research Hospital. Patients, who were admitted to IMCU between April 2019 and January 2021, were analyzed retrospectively. The data of 140 individuals were collected from the hospital database. Chest computer tomography severity score (CT-SS) was used to evaluate the parenchymal involvement. Patients were classified into 2 groups those with mild/moderate parenchymal involvement and those with severe parenchymal involvement. Only laboratory-confirmed cases that were hospitalized in IMCU were included in the analysis. On admission and at discharge, blood parameters including complete blood count (CBC), albumin, C-reactive protein (CRP), procalcitonin, fibrinogen, troponin, and D-dimer values were analyzed. Blood parameters at the time of admission were compared between the patients who died in the IMCU and the others. Age, gender, comorbid disease, the severity of lung involvement, and noninvasive ventilation requirement were analyzed in these two groups. Blood parameters at the time of discharge were compared between survived and deceased patients. The flowchart of the study is shown in Figure 1. Patients who were discharged from IMCU were followed up until November 2021. Postdischarge survival (PDS) was defined as the length of time from discharge to death from any cause. Overall survival (OS) was defined as the time from diagnosis of COVID-19 until death from any cause.

### 2.3. Statistical Analysis

Statistical analysis was performed using the IBM SPSS (Statistical Package for the Social Sciences) Statistics 25.0 Program. Data were expressed in mean ± standard deviation (SD) or median (min-max) for continuous variables and in number *n* (%) for categorical variables. Normality was assessed using the Shapiro–Wilk (*n* < 50) and Kolmogorov–Smirnov tests. The Mann–Whitney test was utilized if the normal distribution was not appropriate. In case normal distribution was appropriate, independent-sample *t*-test was used for the comparison of numerical variables. Categorical variables were assessed using the chi-square test. Survival analysis was performed using the Kaplan–Meier analysis and Cox regression analysis. Numerical variables predicting mortality and noninvasive mechanical ventilation requirement were determined by univariate and multivariate Cox regression analysis. A *p* value of <0.05 was considered statistically significant. The data that support the findings of this study are available from the corresponding author (B.H.) upon reasonable request.

## 3. Results

### 3.1. Sociodemographic and Clinical Characteristics

A total of 140 patients were included within the scope of this study. The median age was 72.5 years, and 77 (55%) of them were male and 63 (45%) of them were female. A total of 37 (26.4%) patients deceased in IMCU, and 40 patients (28.5%) were transferred to ICU. The majority of the patients (*n* = 117, 83.5%) had lung involvement at admission. The severity of lung involvement was as follows: 40.7% (*n* = 57) was mild to moderate and 42.8% (*n* = 60) was severe. Hypertension (HT) (*n* = 84, 60%) was the most common comorbid disease in patients followed by coronary artery disease (CAD) (*n* = 57, 43%), diabetes mellitus (DM) (*n* = 46, 32.9%), cancer (*n* = 31, 22.1), chronic renal failure (CRF) (*n* = 29, 20.7%), pulmonary disease (PD) (*n* = 20, 14.3%), and cerebrovascular disease (CVD) (*n* = 14, 10%). No statistically significant differences have been achieved in terms of age (*p*=0.071), gender (*p*=0.48), the severity of lung involvement (*p*=0.23), and any of comorbidities (HT [*p*=0.63], CAD [*p*=0.52], DM [*p*=0.27], cancer [*p*=0.58], CRF [*p*=0.31], PD [*p*=0.49], and CVD [*p*=0.91]) between patients who died in IMCU and the others. Demographic, clinical characteristics, and laboratory findings of the study population are shown in Table 1.

### 3.2. Respiratory Support

Forty-five patients (32.1%) required masks with reservoir bags, and 30 patients (21.4%) were treated with high-flow nasal cannula oxygen therapy while 24 individuals (17.1%) did not require any respiratory support. Forty-one (29.2%) patients required a noninvasive mechanical ventilator (NIMV) during IMCU hospitalization, and 12 of them (8.6%) had deceased in IMCU, and 18 (12.8%) in ICU while 11 of them (7.8%) survived (Table 2). There was no statistically significant difference in NIMV requirement between patients who died in IMCU and the others (*p*=0.35). In univariate Cox regression analysis, no statistically significant difference has been observed in NIMV requirement between patients who had deceased in IMCU and the others (HR 1.394; 95% CI 0.689–2.818; *p*=0.355). A statistically significant difference was found between the patients who had deceased and survived after discharge (HR 2.306; 95% CI 1.276–4.167; *p*=0.006) (Tables 3 and 4). In multivariate Cox regression analysis, noninvasive mechanical ventilation requirement (HR 2.363; 95% CI 1.201–4.651; *p*=0.013) was associated with an increased risk of mortality at discharge from IMCU (Table 4).

### 3.3. Survival Analysis

**Figure 2:**
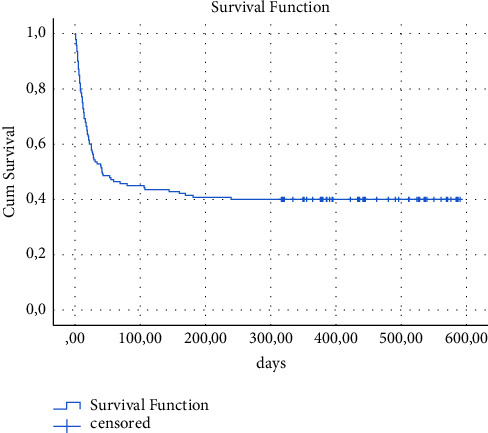
Kaplan–Meier survival analysis by overall survival.

**Figure 3:**
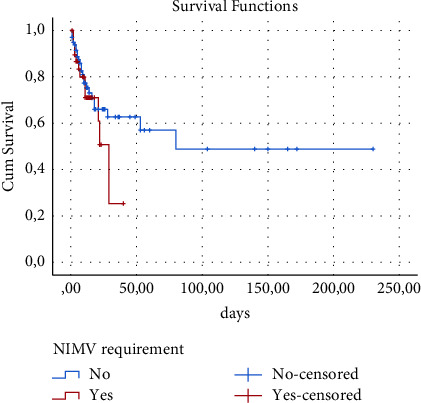
Kaplan–Meier survival analysis by noninvasive mechanic ventilation requirement. Comparison between patients who died in IMCU and the others in NIMV, noninvasive mechanic ventilation requirement.

**Figure 4:**
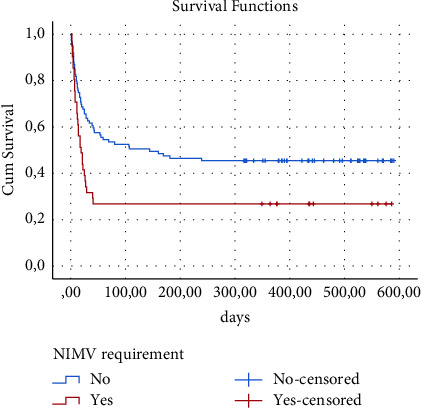
Kaplan–Meier survival analysis by noninvasive mechanical ventilation requirement. Comparison between survivors and nonsurvivors NIMV, noninvasive mechanic ventilation requirement.

A total of 37 patients deceased during IMCU hospitalization. The median length of stay was 6 days (ranging between 1 and 80 days). Out of 40 patients who were transferred to the ICU, 35 (87.5%) had decreased. The median follow-up time in patients who survived after discharge from IMCU was 462 days (ranging between 316 and 590 days), and 12 (19%) discharged patients had died (Table 5). Median PDS in patients who died after discharge from IMCU was 12 days. The overall survival rate was found as 40% while the IMCU survival rate was 73.6%. The median overall survival was 41 days (Figure 2). Median OS was 29 days in patients (*n* = 12) who required NIVM and had deceased in IMCU (Figure 3). Median OS was 19 days in patients (*n* = 41) who required NIVM while 144 days in others (*n* = 99) who did not (*p*=0.008) (Table 2 and Figure 4).

### 3.4. Parameters Predicting Mortality

Higher levels of leukocyte (*p* < 0.001), neutrophil-lymphocyte ratio (NLR) (*p*=0.003), CRP (*p*=0.039), PCT (*p* < 0.001), D-dimer (*p*=0.017), fibrinogen (*p*=0.016), and lower level of albumin (*p*=0.007) were found to be associated with an increased risk of mortality in IMCU regarding the results of Kaplan– Meier analysis, laboratory findings on admission (Table 1). At discharge from IMCU, higher levels of leukocyte (*p* < 0.001), neutrophil (*p* < 0.001), NLR (*p* < 0.001), CRP (*p* < 0.001), PCT (*p* < 0.001), D-dimer (*p*=0.001), and troponin (*p*=0.006) and lower level of hemoglobin (*p*=0.001), hematocrit (*p*=0.006), lymphocyte (*p* < 0.001), lymphocyte monocyte ratio (LMR) (*p*=0.003), platelet (*p*=0.008), and albumin were found to be associated with an increased risk of mortality (Table 6). In univariate analysis, platelet value higher than 400 × 10^3^/*µ*L (HR 3.021; 95% CI 1.235–7.395 *P*=0.015), PCT higher than 0.1 µg/L (HR 2.632; 95% CI 1.021–6.786 *P* = 0.045), and peripheral oxygen saturation ≤90% (HR 3.419; 95% CI 1.740–6.716 *P* < 0.001) were found to be associated with a higher risk of mortality in IMCU (Table 3). In univariate analysis at discharge leukocytes higher than 10.6 × 10^3^/*µ*L (HR 2.168; 95% CI 1.211–3.881 *P*=0.009), neutrophils higher than 6.9 × 10^3^/*µ*L (HR 2.079; 95% CI 1.163–3.717 *P*=0.014), lymphocyte count lower than 0.6 × 10^3^/*µ*L (HR 5.582; 95% CI 2.986-10.436 *p* < 0.001), platelet lower than 140 × 10^3^/*µ*L (HR 3.913; 95% CI 1.890–8.105 *p* < 0.001) and higher than 400 × 10^3^/*µ*L (HR 3.522; 95% CI 1.460–8.494 *P*=0.005), albumin lower than 3.5 g/dl (HR 10.451; 95% CI 1.439–75.903 *P*=0.020), PCT higher than 0.1 *µ*g/L (HR; 5.717; 95% CI 2.641–12.375 *P* < 0.001), NIMV requirement (HR 2.306; 95% CI 1.276–4.167 *P*=0.006), higher level of neutrophil/lymphocyte ratio (HR 1.038; 95% CI 1.021–1.055 *P* < 0.001), and monocyte/lymphocyte ratio (HR 1.371; 95% CI 1.178–1.597 *P* < 0.001) were found to be associated with a higher risk of mortality after discharge from IMCU (Table 4). Variables that independently increased the risk of mortality in multivariable Cox proportional hazard analysis could be elaborated as platelet count higher than 400 × 10^3^/*µ*L (HR 3.454; 95% CI 1.383–8.625; *p*=0.008) and PCT higher than 0.1 *µ*g/L (HR 3.083; 95% CI 1.158–8.206; *p*=0.024) and peripheral oxygen saturation ≤90% (HR 4.121; 95% CI 2.018–8.414; *p* < 0.001) (Table 3). Regarding findings at the time of discharge, platelets lower than 140 × 10^3^/*µ*L (HR 2.269; 95% CI 1.012–5.085; *p*=0.047), PCT higher than 0.1 *µ*g/L (HR 2.809; 95% CI 1.216–6.487; *p*=0.016), and noninvasive mechanic ventilation requirement (HR 2.363; 95% CI 1.201–4.651; *p*=0.013) were found to be associated with a higher risk of mortality after discharge from IMCU (Table 4).

## 4. Discussion

IMCU may contribute to a decrease in mortality by providing closer follow-up and better patient care for patients who need more care than compared to standard clinical treatment but do not require ICU admission [6, 7]. Studies on IMCU in COVID-19 patients have been conducted previously; however, our study was the first article from Turkey. The difference between this research compared to other studies in terms of evaluating hospitalization and discharge parameters separately, long-term follow-up after discharge from IMCU, and evaluation of survival.

Advanced age was found to be associated with increased mortality [11–14]. In a recent IMCU study of 253 patients, Cuartin et al. reported that significant differences have been observed in individuals (65 years and older) with chronic kidney and respiratory diseases in terms of survival analysis. These parameters were defined as independent risk factors for mortality [15]. In our study, age and comorbid diseases were not statistically significant in survival. This situation might be due to the fact that a significant proportion of our cohort was at an advanced age and had the comorbid disease. In our study, the median length of stay in IMCU was found to be 6 days (ranging between 1 and 80), and this result was similar to the Intermediate Respiratory Intensive Care Unit (RICU) study with an average length of stay of 3.3 ± 2.8 days in the deceased and 6.4 ± 3.3 days in the survivors [16].

In previous literature, it was stated that some indicators such as highly sensitive CRP, PCT, creatinine, LDH, AST, hypersensitive cardiac troponin I, prothrombin time, D-dimer, and lymphocyte count are helpful in evaluating the severity of the disease and can predict the prognosis of COVID-19 [4–6]. CRP is usually elevated in some diseases with chronic inflammation such as many cancers. Furthermore, PCT appears to provide better sensitivity and specificity than CRP (respectively, 89% and 94% vs. 71% and 78% for CRP) [17]. In a study examining the risk factors of COVID-19 infection in diabetic patients, PCT elevation was found to be associated with the severity of the infection. In our study cohort, elevated PCT was shown to increase the risks of systemic infection and sepsis in diabetic patients with COVID-19 infection [18]. In our study, diabetes patients constituted a significant portion of approximately one-third (32.9%) of the cohort. More than 25% of COVID-19 patients were diagnosed with Euthyroid sick syndrome (ESS) in another study. COVID-19 patients with ESS had a significantly higher prevalence of severe events and had stronger inflammatory responses, with higher levels of CRP and erythrocyte sedimentation rate as well as a higher positive rate of procalcitonin. Nonetheless, no significant effects of ESS were found on the rates of mortality [19]. In another study, particularly interleukin-6 and procalcitonin were robustly associated with an increased risk of death during hospitalization and reduced hospital discharge [20].

However, PCT may be falsely increased in some neoplastic situations. Thus, some solid tumors (medullar carcinoma of the thyroid and small-cell lung cancer) as well as some hematological malignancies are thought to be associated with PCT positivity [21–24]. In our study cohort, 22.1% of the patients had cancer. There was no difference in mortality between those with and without cancer. Therefore, the cause of high procalcitonin levels, which we found to be associated with increased mortality, was not thought to be related to cancer. In light of the studies mentioned above, although PCT has variable results on mortality in COVID-19 infection, it was shown to increase mortality in our study. The most important reason for this is that the patients are critical intensive care patients and may be considered as being at risk for secondary infections and associated sepsis.

In a national multicenter retrospective study in China, the incidence of thrombocytopenia (<150 × 10^9^/L) was found to be 36.2% in COVID-19, and it was shown that thrombocytopenia was associated with disease severity and mortality [25]. Thrombocytopenia can be explained by 3 different mechanisms. First, systemic inflammation or high IL-6 levels can cause a cytokine storm and suppress the hematopoietic microenvironment and hematopoiesis [26, 27]. Second, SARS-CoV-2 can directly infect hematopoietic stem cells and megakaryocytes through angiotensin-converting enzyme 2 (ACE2), CD13, or CD66a [28]. The third can be evaluated as the presence of antiviral antibodies that crossreact with hematopoietic cells and (or) platelets. Chen et al. showed that patients with COVID-19 have delayed-phase thrombocytopenia as a result of impaired maturation of megakaryocytes [29]. Thrombotic microangiopathy and disseminated intravascular coagulation were demonstrated in autopsies of patients who developed thrombocytopenia and died [30]. In our study, the presence of thrombocytopenia at the time of discharge from IMCU was associated with increased mortality. This may be a component of disseminated intravascular coagulation or a part of bone marrow depression secondary to infection.

Thromboembolic events might lead to sudden death in COVID-19 patients [31]. Platelets play a very important role in thrombogenesis. Recently, it has been shown that high platelet activation, including platelet adhesion and aggregation, *α* granule secretion, and intense granule release, was closely linked to thrombosis in COVID-19. In addition, Zhang et al. showed that the spike protein of SARS-CoV-2 directly stimulates platelets, inducing the release of clotting factors, and thrombosis by increasing inflammatory cytokines and leukocyte-platelet aggregates [32]. In our study, thrombocytosis was found to be associated with increased mortality in IMCU. Based on these data, early thrombocytosis and late thrombocytopenia can be explained as important indicators of increased mortality.

In our study, while the need for noninvasive mechanical ventilation in the critically IMCU had no effect on mortality, postdischarge mortality was found to be higher in patients who required longer mechanical ventilation. On the other hand, noninvasive mechanical ventilation, a mask with a reservoir, and a high-flow nasal cannula can reduce the rate of endotracheal intubation in critical IMCU. In an IMCU study of COVID-19 patients with severe respiratory failure, the endotracheal intubation rate was found to be 37.1% [33]. Among COVID-19 patients hospitalized in critical intensive care units in China, the percentage of those who required ICU ranged from 5% to 32% [1]. In our study, 40 patients were transferred to ICU, and the endotracheal intubation rate was found to be 28.5%. The reason for low intubation rate was thought to be due to the fact that the rate of patients with SpO2 ≤90% was 30%, and early intervention has been performed in hypoxia. In a study conducted in Wuhan, mortality rates were reported between 81% and 97% in patients requiring invasive mechanical ventilation [34]. Similarly, in our study, 35 of 40 patients who required invasive mechanical ventilation and were transferred to the ICU had deceased, and the mortality rate was 87.5%. The reason for higher mortality rate could be explained as the risk of widespread ICU death, intubation, and antibiotic-resistant bacterial superinfections. In the study that included 253 patients mentioned above, 80 patients died during hospitalization, and the mortality rate was 31.6%. The IMCU mortality rate was found to be 24.2% [15]. Similarly, in our study, while the IMCU mortality rate was 26.4%, the mortality rate during hospitalization was found to be 51.4%, which was higher than the aforementioned study.

In our study, low oxygen levels were associated with a high mortality on admission to IMCU. In previous studies, it has been shown that dyspnea during hospitalization and the persistence of hypoxia despite oxygen support are independent predictors of mortality in COVID-19 [35, 36]. There are several mechanisms that elaborate the relationship between hypoxia and mortality in COVID-19 as hypoxia increases viral replication and inflammation. In this way, edema and tissue hypoxia occur at the alveolar level, and mortality increases in COVID-19 [37]. Hypoxia may also be associated with pulmonary vasoconstriction in COVID-19 pneumonia [38]. Under experimental conditions, hypoxia leads to partial protein S deficiency, leading to coagulation [39]. In summary, although hypoxia is a result of viral-induced pulmonary infiltration and pneumonic consolidation, it is thought to increase mortality by causing viral proliferation, lung inflammation, cytokine release, pulmonary vasoconstriction, and intravascular thrombosis in the setting of COVID-19 infection [37].

To access host cells, SARS-CoV-2 uses a surface glycoprotein (peplomer) known as spike; ACE2 has been shown to be a coreceptor for coronavirus entry [40]. ACE2 is also expressed by endothelial cells [41], and other major clinical events usually observed in COVID-19 patients (e.g., high blood pressure [42], thrombosis [43] kidney disease [44], pulmonary embolism [45], cerebrovascular, and neurologic disorders) [46] indicate that the virus is targeting the endothelium, one of the largest organs in the human body [47]. In addition to this, evidence is emerging that the multiorgan injury observed in COVID-19 is a consequence of cytokine-induced endothelial dysfunction (endothelium) [48]. IL-6 causes endothelial activation and neutrophil infiltration, which results in NO (nitric oxide)-mediated changes to vascular permeability and loss of vascular tone [49]. This is reflected clinically by the development of septic shock [43]. Endothelial dysfunction is aggravated by hypoxia, which augments thrombosis by both increasing blood viscosity and hypoxia-inducible transcription factor-dependent signaling pathway [50]. In our study, one of the effects of hypoxia on mortality can be considered as the induction of endothelial damage due to SARS-CoV-2. During the median follow-up of 462 days (ranging between 316 and 590) after discharge from IMCU, 47 of the 103 patients had deceased. Twelve of 63 discharged individuals had died. In a recent study, it was shown that COVID-19 patients developed endothelial dysfunction, which was significantly impaired compared to the healthy control group at 6-month follow-up [51]. Therefore, endothelial dysfunction may be the cause of increased mortality in long-term follow-up after COVID-19, as well as in acute COVID-19 infection. In COVID-19, patients with severe symptoms, as myeloid-derived suppressed cells (MDSCs) increase, so does the activity of arginase, the enzyme responsible for metabolizing L-Arginine into ornithine and urea, and consequently L-Arginine level decreases [52]. Previous research has shown that reduced L-Arginine levels increase the production of reactive oxygen species (ROS) and exacerbate inflammation in the endothelium [53]. Arginine is an amino acid that acts as a substrate for endothelial nitric oxide (NO) synthase (eNOS). It has been previously shown to significantly improve endothelial function, providing a strong rationale for its use in COVID-19 patients [54]. Several investigators have suggested that endotheliitis may be the critical mechanism underlying the impaired systemic microcirculatory function observed in various vascular beds in patients experiencing prolonged COVID-19 symptoms. Consistent with this, the Lincoln survey indicates that the supplementation with l-Arginine + Vitamin C has beneficial effects on Long-COVID, in terms of attenuating its typical symptoms and improving effort perception [55]. Based on these data, L-Arginine, which has been shown to improve endothelial functions and reduce prolonged COVID-19 symptoms, may improve survival in long-term follow-up after COVID-19 infection. On the other hand, the advanced age and comorbidity of the patients admitted to IMCU should also be considered, regardless of the COVID-19 infection, as mortality is already high in this group of patients. The limitation of our study could be elaborated as a single-center retrospective study and the exact causes of death after hospital discharge could not be determined.

## 5. Conclusions

IMCU can contribute to the multidisciplinary management of the disease by providing noninvasive respiratory support and close monitoring to COVID-19 patients and can reduce ICU occupancy rates. Based on the risk factors demonstrated in previous studies and our findings, a risk scoring system should be developed for COVID-19 patients admitted to IMCU. In addition, early transfer to the ICU in patients with high-risk factors may improve survival outcomes. Multicenter prospective studies are needed to confirm this proposition. IMCU units are important in terms of contributing to reducing ICU bed occupancy rates and reducing mortality in COVID-19 patients.

In light of the results of our study, high platelet count and procalcitonin levels, hypoxia was associated with an increased risk of mortality in IMCU. At discharge from IMCU low platelet count, high procalcitonin levels and the requirement for noninvasive mechanical ventilation were found to be associated with increased overall mortality. It could be stated that monitoring of these parameters in the follow-up of COVID-19 patients might enhance treatment outcomes via reducing mortality and morbidity.

## Figures and Tables

**Table 1:** Kaplan–Meier analysis parameters predicting IMCU mortality.

	All patients (*n* = 140)	Survived (*n* = 103)	Death (*n* = 37)	*P* value
Age, year	71.27 ± 12.36	68.96 ± 13	72.8 ± 11.64	0.071
Gender				
(i) Male, *n* (%)	77 (55)	54 (70.1)	23 (29.9)	0.48
(ii) Female, *n* (%)	63 (45)	49 (77.8)	14 (22.2)	
Comorbidities, *n* (%)				
(i) Hypertension	84 (60)	63 (75)	21 (25)	0.63
(ii) Coronary artery disease	57 (43)	43 (75.4)	14 (24.6)	0.52
(iii) Diabetes mellitus	46 (32.9)	37 (80.4)	9 (19.6)	0.27
(iv) Cancer	31 (22.1)	20 (64.5)	11 (35.5)	0.58
(v) Chronic renal failure	29 (20.7)	19 (65.5)	10 (34.5)	0.31
(vi) COPD/pulmonary disease	20 (14.3)	13 (65)	7 (35)	0.49
(vii) Cerebrovascular disease	14 (10)	10 (71.4)	4 (28.6)	0.91
Lung parenchymal involvement				
(i) Mild to moderate	57	42 (73.7)	15 (26.3)	0.23
(ii) Severe	60	43 (71.7)	17 (28.3)	
Noninvasive ventilation requirement *n* (%)	41 (29.2)	29 (20.7)	12 (8.5)	0.36
SpO_2_ ≤90%	52 (30)			
Blood tests on admission				
WBC (/*µ*L) × 10^9^	9.6 (0.2–38.8)	8.2 (0.6–24.8)	12.7 (0.2–38.8)	<0.001
Neutrophil (/*µ*L) × 10^9^	7.9 (0.3–36.3)	6.8 (0.3–16.2)	10.5 (10–36.3)	0.062
Lymphocyte (/*µ*L) × 10^9^	0.7 (0.1–17.8)	0.7 (0.2–17.8)	0.8 (0.1–3.7)	0.27
Neutrophil/lymphocyte	9.29 (0.1–73.9)	9.28 (0.6–73.9)	9.5 (0.1–49)	0.003
Monocytes (/*µ*L) × 10^9^	0.8 (0.02–14.7)	0.5 (0.1–14.7)	0.6 (0.02–9.5)	0.552
Lymphocyte/monocytes	1.6 (0.09–34)	1.5 (0.09–34)	1.5 (0.28–24)	0.84
Hemoglobin (g/dl)	11 (5.7–16.5)	11.3 (5.7–16.5)	10.9 (6.3–15.5)	0.36
Hematocrit (%)	33.1 (17.1–53.2)	33.9 (17.1–53.2)	32 (18.3–49.8)	0.36
Platelet (/*µ*L) × 10^9^	243 (7–867)	225 (12–867)	247 (7–810)	0.31
CRP (mg/L)	105 (2–400)	102 (2–400)	129.5 (8.7–372.7)	0.039
PCT (*µ*g/L)	0.78 (0.01–75)	0.18 (0.01–51.37)	0.95 (0.02–75)	<0.001
Albumin (g/dl)	3 (1.3–3.4)	3.1 (1.3–3.2)	2.8 (1.9–3.4)	0.007
D-dimer (*µ*g/L)	1880 (240–26820)	1310 (240–14310)	2380 (540–26820)	0.017
Troponin (ng/mL)	60 (2.8–44026)	27.8 (3.2–44026)	40.4 (2.8–2780)	0.549
Fibrinogen (mg/dl)	655 (185.8–3315)	494 (185.8–3315)	714.3 (236.8–900)	0.016

Data are shown as number (percentage (n (%)) and median (minimum-maximum). COPD, chronic obstructive pulmonary disease; WBC, white blood cells; CRP, C-reactive protein; PCT, procalcitonin.

**Table 2:** Percentage of death and survival in patients who required NIVM.

Noninvasive ventilation requirement (*n* = 41) (29.2%)				
Survived	*n* (%)	Death	*n* (%)	*P* value
Total	11 (7.8)	Total	30 (21.4)	0.008
Patients who were discharged to home	9 (6.4)	Death in IMCU	12 (8.6)	
Patients who were transferred to ICU	2 (1.4)	Death in ICU	18 (12.8)	

**Table 3:** Univariate and multivariate Cox regression analysis and parameters on admission predicting mortality.

Cox regression analysis								
	Univariate				Multivariate			
	95% CI				95% CI			
Parameters	HR	Lower	Upper	*P*value	HR	Lower	Upper	*P*value
WBC (*µ*L) >10.6 × 10^3^	1.778	0.923	3.423	0.085	—	—	—	—
Neutrophil (*µ*L) >6.9 × 10^3^	1.385	0.717	2.676	0.332	—	—	—	—
Lymphocyte (*µ*L) <0.6 × 10^3^	0.735	0.355	1.521	0.407	—	—	—	—
Monocyte (*µ*L) >0.9 × 10^3^	1.101	0.528	2.297	0.797	—	—	—	—
NLO	1.015	0.994	1.037	0.157	—	—	—	—
MLO	0.939	0.769	1.146	0.534	—	—	—	—
Hemoglobin (gr/dl) <14.1	1.208	0.370	3.943	0.754	—	—	—	—
Hematocrit <43.3%	1.019	0.312	3.334	0.975	—	—	—	—
Platelet (*µ*L) <140 × 10^3^	1.642	0.671	4.018	0.278	—	—	—	—
Platelet (*µ*L) >400 × 10^3^	3.021	1.235	7.395	0.015	**3.454**	**1.383**	**8.625**	**0.008**
Albumin (g/dl) <3.5	21.679	0.002	213 × 10^3^	0.512	—	—	—	—
D-dimer (*µ*g/L) >440	20.658	0.001	897 × 10^4^	0.648	—	—	—	—
Fibrinogen (mg/dl) >420	2.959	0.674	12.986	0.151	—	—	—	—
CRP (mg/L) >5	20.450	0.001	324 × 10^6^	0.721	—	—	—	—
Troponin (ng/L) >46	1.100	0.521	2.324	0.802	—	—	—	—
PCT (*µ*g/L) >0.1	2.632	1.021	6.786	0.045	**3.083**	**1.158**	**8.206**	**0.024**
NIMV	1.394	0.689	2.818	0.355	—	—	—	—
SpO_2_ ≤90%	3.419	1.740	6.716	<0.001	**4.121**	**2.018**	**8.414**	**<0.001**

CI, confidence interval; HR, hazard ratio; WBC, white blood cells; NLO, neutrophil/lymphocyte; MLO, monocyte/lymphocyte; CRP, C-reactive protein; PCT, procalcitonin; NIMV, noninvasive mechanic ventilation requirement; SpO2, peripheral oxygen saturation. Bold values denote statistical significance at the *p* < 0.05 level.

**Table 4:** Univariate and multivariate Cox regression analysis and parameters at the time of discharge predicting mortality.

Cox regression analysis								
	Univariate				Multivariate			
	95% CI				95% CI			
Parameters	HR	Lower	Upper	*P*value	HR	Lower	Upper	*P*value
WBC (*µ*L) >10.6 × 10^3^	2.168	1.211	3.881	0.009	—	—	—	—
Neutrophil (*µ*L) >6.9 × 10^3^	2.079	1.163	3.717	0.014	—	—	—	—
Lymphocyte (*µ*L) <0.6 × 10^3^	5.582	2.986	10.436	<0.001	—	—	—	—
Monocyte (*µ*L) >0.9 × 10^3^	1.264	0.654	2.442	0.485	—	—	—	—
NLO	1.038	1.021	1.055	<0.001	1.019	0.998	1.041	0.073
MLO	1.371	1.178	1.597	<0.001	—	—	—	—
Hemoglobin (gr/dl) <14.1	0.820	0.199	3.386	0.784	—	—	—	—
Hematocrit <43.3%	0.923	0.127	6.705	0.937	—	—	—	—
Platelet (*µ*L) <140 × 10^3^	3.913	1.890	8.105	<0.001	**2.269**	**1.012**	**5.085**	**0.047**
Platelet (*µ*L) >400 × 10^3^	3.522	1.460	8.494	0.005	2.042	0.823	5.066	0.124
Albumin (g/dl) <3.5	10.451	1.439	75.903	0.020	5.650	0.760	42.002	0.091
D-dimer (*µ*g/L) >440	1.615	0.391	6.674	0.508	—	—	—	—
Fibrinogen (mg/dl) >420	1.312	0.612	2.812	0.485	—	—	—	—
CRP (mg/L) >5	2.849	0.392	20.686	0.301	—	—	—	—
Troponin (ng/L) >46	1.357	0.692	2.662	0.375	—	—	—	—
PCT (*µ*g/L) >0.1	5.717	2.641	12.375	<0.001	**2.809**	**1.216**	**6.487**	**0.016**
NIMV requirement	2.306	1.276	4.167	0.006	**2.363**	**1.201**	**4.651**	**0.013**
SpO_2_ ≤90%	1.788	0.984	3.249	0.056	—	—	—	—

CI, confidence interval; HR, hazard ratio; WBC, white blood cells; NLO, neutrophil/lymphocyte; MLO, monocyte/lymphocyte; CRP, C-reactive protein; PCT, procalcitonin; NIMV, noninvasive mechanic ventilation; SpO_2_, peripheral oxygen saturation. Bold values denote statistical significance at the *p* < 0.05 level.

**Table 5:** Percentage of death and survival in all patients.

Patients who died in IMCU *n*(%)	37 (26.4)
Patients who were transferred to ICU	
(i) Total *n* (%)	40 (28.5)
(ii) Survived *n* (%)	5 (3.5)
(iii) Death *n* (%)	35 (25)
Patients who were discharged to home	
(i) Total *n* (%)	63 (45)
(ii) Survived *n* (%)	51 (36.5)
(iii) Death *n* (%)	12 (8.6)

**Table 6:** Kaplan–Meier analysis and blood parameters predicting mortality after discharge.

Blood tests at the time of discharge	All patients (*n* = 103)	Survived (*n* = 56)	Death (*n* = 47)	*P* value
WBC (/*µ*L) × 10^9^	9.5 (0.1–37.7)	7.6 (4.3–37.7)	11.3 (0.1–37.4)	<0.001
Neutrophil (/*µ*L) × 10^9^	6 (0.01–35)	5.6 (1.6–13.6)	8.9 (0.01–35)	<0.001
Lymphocyte (/*µ*L) × 10^9^	1.1 (0.1–29.7)	1.55 (0.16–29.7)	0.7 (0.1–4.1)	<0.001
Neutrophil/lymphocyte	6.5 (0.22–70)	4 (0.22–35.6)	11.5 (2.2–70)	<0.001
Monocytes,(/*µ*L) × 10^9^	0.68 (0.2–6.4)	0.7 (0.2–1.6)	0.5 (0.3–6.4)	0.154
Lymphocyte/monocytes	1.9 (0.08–49.5)	2 (0.23–49.5)	1.5 (0.08–13.3)	0.003
Hemoglobin (g/dl)	10.8 (6.6–16.1)	10.9 (7.5–15.1)	9.6 (6.6–16.1)	0.001
Hematocrit (%)	30 (19.8–48)	32.2 (22.2–45)	29.1 (19.8–48)	0.006
Platelet (/*µ*L) × 10^9^	255 (4–619)	258 (124–590)	214.5 (4–619)	0.008
CRP (mg/L)	67.6 (0.1–520)	28 (0.1–273)	145.8 (0.6–520)	<0.001
PCT (*µ*g/L)	0.7 (0–121.3)	0.07 (0–12.8)	0.75 (0.01–121.3)	<0.001
Albumin (g/dl)	2.8 (1.4–4)	3.15 (1.7–4)	2.5 (1.4–3.5)	<0.001
D-dimer (*µ*g/L)	2150 (240–17480)	1260 (240–16400)	2490 (290–17480)	<0.001
Troponin (ng/L)	65 (2.5–22317)	24 (2.5–6315)	66 (3.8–22317)	0.006
Fibrinogen (mg/dl)	536 (69–914)	466.6 (69–893)	547.6 (208.1–914)	0.2

Data are shown as median (minimum-maximum). WBC, white blood cells; CRP, C-reactive protein; PCT, procalcitonin.

## Data Availability

Necessary data can be obtained from the corresponding author.

## References

[1] Guan W. J., Ni Z. Y., Hu Y. (2020). Clinical characteristics of coronavirus disease 2019 in China. *New England Journal of Medicine*.

[2] Wilson N., Kvalsvig A., Barnard L. T., Baker M. G. (2020). Case-fatality risk estimates for COVID-19 calculated by using a lag time for fatality. *Emerging Infectious Diseases*.

[3] Wu Z., McGoogan J. M. (2020). Characteristics of and important lessons from the coronavirus disease 2019 (COVID-19) outbreak in China: summary of a report of 72 314 cases from the Chinese Center for Disease Control and Prevention. *Journal of the American Medical Association*.

[4] Emanuel E. J., Persad G., Upshur R. (2020). Fair allocation of scarce medical resources in the time of COVID-19. *New England Journal of Medicine*.

[5] Carenzo L., Costantini E., Greco M. (2020). Hospital surge capacity in a tertiary emergency referral centre during the COVID-19 outbreak in Italy. *Anaesthesia*.

[6] Capuzzo M., Volta C., Tassinati T. (2014). Hospital mortality of adults admitted to Intensive Care Units in hospitals with and without Intermediate Care Units: a multicentre European cohort study. *Critical Care*.

[7] Lavina L. W. M., Fumeaux T. (2017). Intermediate care units: zwischen ICU und bettenstation. *Schweiz Arztzeitg*.

[8] Liao D., Zhou F., Luo L. (2020). Haematological characteristics and risk factors in the classification and prognosis evaluation of COVID-19: a retrospective cohort study. *The Lancet Haematology*.

[9] Zheng Z., Peng F., Xu B. (2020). Risk factors of critical & mortal COVID19 cases: a systematic literature review and meta-analysis. *Journal of Infection*.

[10] Henry B. M., de Oliveira M. H. S., Benoit S., Plebani M., Lippi G. (2020). Hematologic, biochemical and immune biomarker abnormalities associated with severe illness and mortality in coronavirus disease 2019 (COVID-19): a meta-analysis. *Clinical Chemistry and Laboratory Medicine*.

[11] Zhang L., Yan X., Fan Q. (2020). D-dimer levels on admission to predict inhospital mortality in patients with COVID-19. *Journal of Thrombosis and Haemostasis*.

[12] Li X., Xu S., Yu M. (2020). Risk factors for severity and mortality in adult COVID-19 inpatients in Wuhan. *Journal of Allergy and Clinical Immunology*.

[13] Du R. H., Liang L. R., Yang C. Q. (2020). Predictors of mortality for patients with COVID-19 pneumonia caused by SARS–CoV2: a prospective cohort study. *European Respiratory Journal*.

[14] Imam Z., Odish F., Gill I. (2020). Older age and comorbidity are independent mortality predictors in a large cohort of 1,305 COVID-19 patients in Michigan, United States. *Journal of Internal Medicine*.

[15] Wu C., Chen X., Cai Y. (2020). Risk factors associated with acute respiratory distress syndrome and death in patients with coronavirus disease 2019 pneumonia in Wuhan, China. *Journal of American Medical Association Internal Medicine*.

[16] Carpagnano G. E., Migliore G., Grasso S. (2021). More skilled clinical management of COVID-19 patients modified mortality in an intermediate respiratory intensive care unit in Italy. *Respiratory Research*.

[17] Muller B., Becker K. L., Schächinger H. (2000). Calcitonin precursors are reliable markers of sepsis in a medical intensive care unit. *Critical Care Medicine*.

[18] Zhang N., Wang C., Zhu F. (2020). Risk factors for poor outcomes of diabetes patients with COVID-19: a single-center, retrospective study in early outbreak in China. *Frontiers in Endocrinology*.

[19] Zou R., Wu C., Zhang S. (2020). Euthyroid sick syndrome in patients with COVID-19. *Frontiers in Endocrinology*.

[20] Kaltoft M., Glavind K. S., Nielsen S. F. (2022). Lipoprotein(a) during COVID-19 hospitalization: thrombosis, inflammation, and mortality. *Atherosclerosis*.

[21] Sira L., Balogh Z., Vitális E. (2021). Case report: medullary thyroid cancer workup initiated by unexpectedly high procalcitonin level-endocrine training saves life in the COVID-19 unit. *Frontiers in Endocrinology*.

[22] Carnino L., Betteto S., Loiacono M. (2010). Procalcitonin as a predictive marker of infections in chemoinduced neutropenia. *Journal of Cancer Research and Clinical Oncology*.

[23] Bihan H., Becker K. L., Snider R. H. (2003). Calcitonin precursor levels in human medullary thyroid carcinoma. *Thyroid*.

[24] Cate C. C., Pettengill O. S., Sorenson G. D. (1986). Biosynthesis of procalcitonin in small cell carcinoma of the lung. *Cancer Research*.

[25] Suarez-Cuartin G., Gasa M., Bermudo G. (2021). Clinical outcomes of severe COVID-19 patients admitted to an intermediate respiratory care unit. *Frontiers of Medicine*.

[26] Yang X., Yang Q., Wang Y. (2020). Thrombocytopenia and its association with mortality in patients with COVID-19. *Journal of Thrombosis and Haemostasis*.

[27] Valletta S., Thomas A., Meng Y. (2020). Micro-environmental sensing by bone marrow stroma identifies IL-6 and TGF*β*1 as regulators of hematopoietic ageing. *Nature Communications*.

[28] Amgalan A., Othman M. (2020). Exploring possible mechanisms for COVID-19 induced thrombocytopenia: unanswered questions. *Journal of Thrombosis and Haemostasis*.

[29] Chen W., Li Z., Yang B. (2020). Delayed-phase thrombocytopenia in patients with coronavirus disease 2019 (COVID-19). *British Journal of Haematology*.

[30] Levi M., Thachil J., Iba T., Levy J. H. (2020). Coagulation abnormalities and thrombosis in patients with COVID-19. *The Lancet Haematology*.

[31] Bikdeli B., Madhavan M. V., Jimenez D. (2020). COVID-19 and thrombotic or thromboembolic disease: implications for prevention, antithrombotic therapy, and follow-up. *Journal of the American College of Cardiology*.

[32] Zhang S., Liu Y., Wang X. (2020). SARS–CoV2 binds platelet ACE2 to enhance thrombosis in COVID-19. *Journal of Hematology & Oncology*.

[33] Rubia J. C. H., Sanchez-Carpintero A. M., Yordi L. A. (2020). Outcomes of an Intermediate Respiratory Care Unit in the COVID-19 Pandemic. *PLOS ONE*.

[34] Weiss P., Murdoch D. R. (2020). Clinical course and mortalite risk of severe COVID-19. *Lancet*.

[35] Xie J., Covassin N., Fan Z. (2020). Association between hypoxemia and mortality in patients with COVID-19. *Mayo Clinic Proceedings*.

[36] Kashani K. B. (2020). Hypoxia in COVID-19: sign of severity or cause for poor outcomes. *Mayo Clinic Proceedings*.

[37] Somers V. K., Kara T., Xie J. (2020). Progressive hypoxia: a pivotal pathophysiologic mechanism of COVID-19 pneumonia. *Mayo Clinic Proceedings*.

[38] Wadman M., Couzin-Frankel J., Kaiser J., Matacic C. (2020). How does coronavirus kill? Clinicians trace a ferocious rampage through the body, from brain to toes. https://pesquisa.bvsalud.org/global-literature-on-novel-coronavirus-2019-ncov/resource/pt/covidwho-71625.

[39] Pilli V. S., Datta A., Afreen S., Catalano D., Szabo G., Majumder R. (2018). Hypoxia downregulates protein S expression. *Blood*.

[40] Letko M., Marzi A., Munster V. (2020). Functional assessment of cell entry and receptor usage for SARS–CoV2 and other lineage B betacoronaviruses. *Nat. Microbiol.*.

[41] Lovren F., Pan Y., Quan A. (2008). Angiotensin converting enzyme-2 confers endothelial protection and attenuates atherosclerosis. *American Journal of Physiology - Heart and Circulatory Physiology*.

[42] Schiffrin E. L., Flack J., Ito S., Muntner P., Webb C. (2020). Hypertension and COVID-19. *American Journal of Hypertension*.

[43] Zhou F., Yu T., Du R. (2020). Clinical course and risk factors for mortality of adult inpatients with COVID-19 in Wuhan, China: a retrospective cohort study. *The Lancet*.

[44] Durvasula R., Wellington T., McNamara E., Watnick S. (2020). COVID-19 and kidney failure in the acute care setting: our experience from seattle. *American Journal of Kidney Diseases*.

[45] Rotzinger D. C., Beigelman-Aubry C., von Garnier C., Qanadli S. D. (2020). Pulmonary embolism in patients with COVID-19: time to change the paradigm of computed tomography. *Thrombosis Research*.

[46] Aggarwal G., Lippi G., Michael Henry B. (2020). Cerebrovascular disease is associated with an increased disease severity in patients with Coronavirus Disease 2019 (COVID-19): a pooled analysis of published literature. *International Journal of Stroke*.

[47] Cooke J. P. (2000). The endothelium: a new target for therapy. *Vascular Medicine*.

[48] Varga Z., Flammer A. J., Steiger P. (2020). Endothelial cell infection and endotheliitis in COVID-19. *The Lancet*.

[49] Merad M., Martin J. C. (2020). Pathological inflammation in patients with COVID-19: a key role for monocytes and macrophages. *Nature Reviews Immunology*.

[50] Gupta N., Zhao Y. Y., Evans C. E. (2019). The stimulation of thrombosis by hypoxia. *Thrombosis Research*.

[51] Oikonomou E., Souvaliotis N., Lampsas S. (2022). Endothelial dysfunction in acute and long standing COVID- 19: a prospective cohort study. *Vascular Pharmacology*.

[52] Dean M. J., Ochoa J. B., Sanchez-Pino M. (2021). Transcriptome and functions of granulocytic myeloid-derived suppressor cells determine their association with disease severity of COVID-19. *medRxiv*.

[53] Lucas R., Czikora I., Sridhar S. (2013). Arginase 1: an unexpected mediator of pulmonary capillary barrier dysfunction in models of acute lung injury. *Frontiers in Immunology*.

[54] Adebayo A., Varzideh F., Wilson S. (2021). l-Arginine and COVID-19: an update. *Nutrients*.

[55] Izzo R., Trimarco V., Mone P. (2022). Combining L-Arginine with vitamin C improves long-COVID symptoms: the LINCOLN Survey. *Pharmacological Research*.

